# Giant Cellulitis-Like Sweet Syndrome Masquerading As Cellulitis and Shingles: A Case Report and Literature Review

**DOI:** 10.7759/cureus.36232

**Published:** 2023-03-16

**Authors:** Weiying Li, Arooj Mian, Kashaf Zaidi, Tasnuva Mahmud

**Affiliations:** 1 Internal Medicine, AdventHealth Orlando, Orlando, USA

**Keywords:** acute myeloid leukemia (aml), shingles, giant cellulitis like sweet syndrome, cellulitis, chronic myeloid leukemia, acute febrile neutrophilic dermatosis, sweet syndrome

## Abstract

Sweet syndrome (SS) is also known as acute febrile neutrophilic dermatoses. Clinically, SS features fever, arthralgias, and the sudden onset of an erythematous rash. The morphologies of skin lesions in SS are heterogenous, varying from papules, plaques, and nodules to hemorrhagic bullae, which sometimes makes the diagnosis of SS more challenging. We report a 62-year-old obese male with a history of chronic myeloid leukemia in remission for 10 years who presented with a rash for five days. The patient reported prodromal flu-like symptoms with subjective fever, malaise, cough, and nasal congestion followed by a sudden onset, painful, non-pruritic rash. The rash was associated with bilateral hip arthralgias and abdominal pain. The patient denied any recent travel, exposure to sick contacts, or the use of any new medications. Physical examination showed a well-demarcated, non-blanching, confluent, erythematous plaque involving the bilateral buttocks and extending to the lower back and flanks with coalescent “juicy”-appearing plaques and flaccid bullae. No oral or mucosal involvement was noted. Laboratory investigations revealed mild leukocytosis, elevated inflammatory markers, and acute kidney injury. The patient was started on antibiotics given the cellulitis-like skin lesions, leukocytosis with neutrophilia, and elevated inflammatory markers. Dermatology was consulted, who attributed the patient’s rash to shingles and recommended initiating acyclovir and obtaining a skin biopsy. However, the patient’s rash and arthralgias worsened with anti-viral treatment while awaiting pathology results. Antinuclear antibodies, complement, human immunodeficiency virus, hepatitis panel, blood cultures, and tumor markers were all negative. Flow cytometry showed no evidence of hematopoietic neoplasms. The skin punch biopsy revealed dense neutrophilic infiltration in the dermis with no evidence of leukocytoclastic vasculitis, consistent with acute neutrophilic dermatoses.

The diagnosis of giant cellulitis-like Sweet syndrome was established, and the patient was started on prednisone 60 milligrams daily. His symptoms improved promptly with steroid treatment. Our case suggests that SS can camouflage a wide spectrum of diseases, including cellulitis, shingles, vasculitis, drug eruptions, leukemia cutis, and sarcoidosis, which emphasizes the importance of keeping a high index of suspicion for SS when assessing the clinical constellations of fever, neutrophilia, and erythematous plaques suggesting atypical cellulitis. Approximately 21% of Sweet syndrome is associated with malignancy. Sweet syndrome can precede, concur with, or follow the onset of malignancy. Due to the lack of a systematic approach to patients with SS, under-investigation and diagnostic delays are common. Therefore, further screening and continuous monitoring in patients with SS becomes especially important in facilitating the early detection of a potential underlying malignancy and assists in initiating adequate therapy.

## Introduction

Sweet syndrome (SS), first described by Dr. Robert Douglas Sweet in 1964 [1], is also known as acute febrile neutrophilic dermatoses. Clinically, SS features fever, arthralgia, and sudden onset of erythematous rash. However, skin lesion morphologies are heterogenous, varying from papules, plaques, and nodules to hemorrhagic bullae; occasionally, pseudo-vesicles can be observed due to secondary superficial dermal edema, which sometimes makes the diagnosis of SS more challenging. In our case, the acute onset erythematous plaques with fever and leukocytosis masqueraded as cellulitis [2] - the burning pain associated with the skin rash and the presence of pseudo-vesicles mimicked shingles, so the patient was treated with antibiotics and acyclovir prior to pathological evidence of SS.

## Case presentation

**Figure 1:**
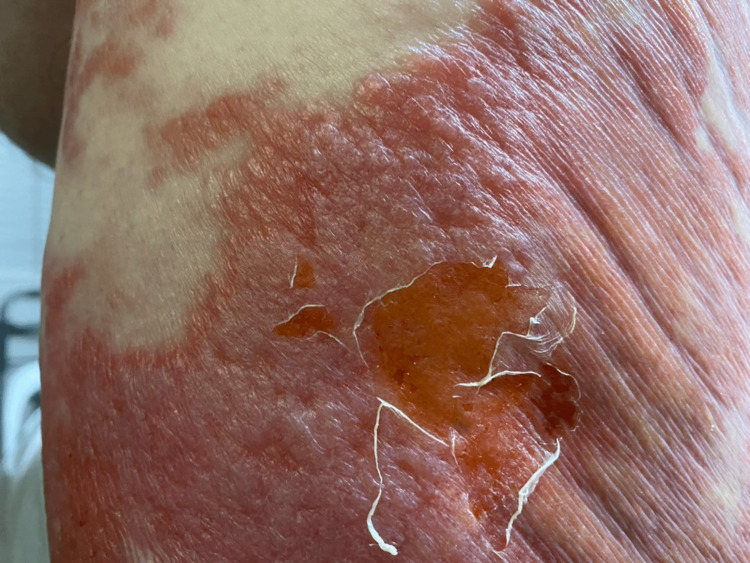
Appearance of the skin eruption A well-demarcated, non-blanching, confluent, erythematous plaque involving the bilateral buttocks and extending to the lower back and flanks with coalescent “juicy”-appearing plaques and flaccid bullae

A 62-year-old obese male with a history of chronic myeloid leukemia (CML) in remission for 10 years presented with a rash for five days. The patient reported prodromal flu-like symptoms with subjective fever, malaise, cough, and nasal congestion followed by a sudden-onset, painful, non-pruritic rash involving the lower back and buttocks. The rash started from the right buttocks and progressed to involve the left buttocks, bilateral lower back, and thighs. It was associated with bilateral hip arthralgias and abdominal pain. The patient denied any recent travel, exposure to sick contacts, or the use of any new medications. On admission, his vital signs were stable. Physical examination showed a well-demarcated, non-blanching, confluent, erythematous plaque involving the bilateral buttocks and extending to the lower back and flanks with coalescent “juicy”-appearing plaques and flaccid bullae (Figure 1). No oral or mucosal involvement was noted.

**Figure 2:**
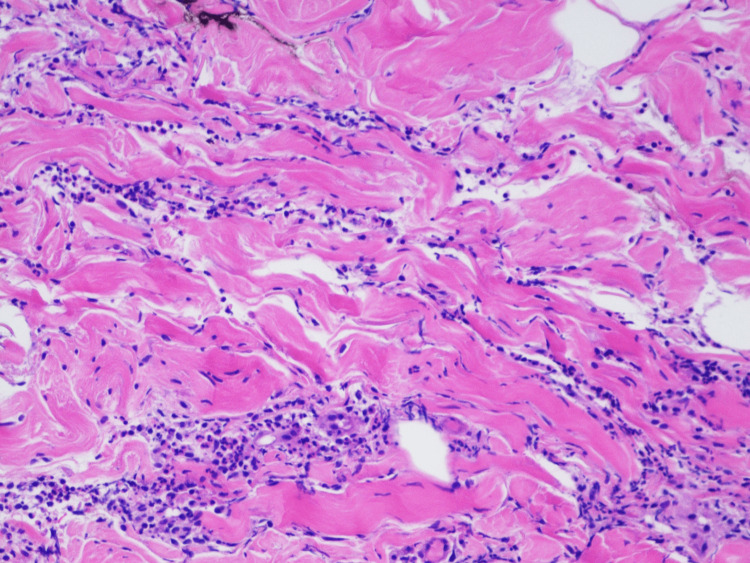
Histologic findings from the skin biopsy Dense infiltrate of neutrophils in the dermis with an absence of vasculitis

Laboratory investigations revealed mild leukocytosis with 91% neutrophils, C-reactive protein (CRP) 246 mg/L, and acute kidney injury. Antinuclear antibodies (ANA), complement, human immunodeficiency virus (HIV), hepatitis panel, blood cultures, and tumor markers (CEA, CA 19-9, CA 125) were all negative. Flow cytometry showed no evidence of hematopoietic neoplasms. The patient was started on antibiotics given the cellulitis-like skin lesions, leukocytosis with neutrophilia, and elevated inflammatory markers. Dermatology was consulted, which attributed the patient’s rash to shingles and recommended initiating acyclovir and obtaining a skin biopsy. However, the patient’s rash and arthralgias worsened with anti-viral treatment while awaiting pathology results. The skin punch biopsy revealed dense neutrophilic infiltration in the dermis with no evidence of leukocytoclastic vasculitis, consistent with acute neutrophilic dermatoses (Figure 2).

The diagnosis of the giant cellulitis-like Sweet syndrome was established and the patient was started on prednisone 60 milligrams daily. His symptoms improved promptly with steroid treatment.

## Discussion

The diagnosis of SS can be challenging since the skin lesions can camouflage a wide spectrum of diseases; other differential diagnoses include shingles, vasculitis, drug eruptions, leukemia cutis, and sarcoidosis. Interestingly, some case reports suggest that cellulitis may trigger the development of SS, and cellulitis and SS may simultaneously coexist. A 38-year-old and a 64-year-old woman developed SS on other parts of the body a few days after developing cellulitis in the lower limbs [3]. The concept of the giant cellulitis-like Sweet syndrome was suggested as a new variant of SS that had been rarely reported. It was uncertain whether the diagnosis of preceding or coexisting cellulitis could be established since SS is also a cellulitis mimicker. A series of case reports showed that five out of eight patients with giant cellulitis-like SS were correlated with various malignancies such as myeloma, breast carcinoma, and acute myeloid leukemia (AML) [4,5]. A rare histologic variant of the crypto-coccoid Sweet syndrome can present with atypical hemorrhagic bullae and yeast-like structures imitating cutaneous cryptococcal disease [6]. The histological finding of a dense neutrophilic infiltrate without leukocytoclastic vasculitis supports the diagnosis of SS, therefore skin biopsy should be performed whenever feasible. However, acute neutrophilic dermatoses are not a unique finding of SS; it is an umbrella term including Sweet syndrome, pyoderma gangrenosum [7], necrotizing neutrophilic dermatoses, Bechet syndrome, and a diverse group of disorders that share similar histological findings of abundant neutrophil infiltration in the absence of infection. Therefore, recognizing the clinicopathological features in the context of the associated diseases and history of prodrome or temporal drug exposure is crucial for establishing the diagnosis.

Traditionally, Sweet syndrome is categorized as classic/idiopathic, malignancy, and drug-associated. Moreover, autoimmune disorders, such as sicca syndrome and primary biliary cholangitis, have been reported as causes of SS as well [8]. The correlation between AML and SS is well-established and supported by a plethora of case reports [9,10]. It is worth noting that certain anti-neoplastic drugs, such as pemetrexed, azacytidine, midostaurin, Enasidenib, and palbociclib can be potential triggers of SS, which complicates the clinical scenario when patients with malignancy develop SS while undergoing chemotherapy and raises a question about the true cause of SS. For example, a patient with AML developed SS one week after starting chemotherapy. It was reported as AML-associated SS; however, it remains questionable whether drug exposure could be the contributing factor [10]. Usually, there is a temporal relationship between drug commencement and disease onset and between drug discontinuation and clinical resolution. Although a skin lesion typically develops two weeks after exposure, it seems that the timeframe between drug exposure and disease onset is highly variable and uncertain. In a case of ipilimumab-induced SS, a patient with melanoma developed SS 19 days after the fourth cycle of ipilimumab infusion [11] while another patient developed SS after the second cycle of ipilimumab exposure [12]; it was not mentioned whether discontinuing the medication resulted in the improvement of symptoms, to prove causality. Other common medications frequently associated with SS include antibiotics such as trimethoprim-sulfamethoxazole, antiseizure medications such as lamotrigine and commonly used drugs such as dapagliflozin, clopidogrel, and dabigatran [13].

The pathogenesis of SS is poorly understood. Many theories were proposed: genetic susceptibility predisposing individuals to cytokine dysregulation upon exposure to certain antigens (which mechanistically explains why SS often occurs with malignancy, pregnancy [14], radiation [15], or new drug exposure), all of which allows the body to react to newly exposed antigens. With the widespread use of the COVID-19 vaccine, more and more cases of COVID-19 vaccine-associated SS have been reported [16], which raises suspicion for an association between the COVID-19 vaccine and SS, however, definite causality has not been established. Increasing evidence shows that granulocytes-colony stimulating factor (G-CSF) plays an important role in the pathogenesis: higher levels of G-CSF detected in patients with active SS; CSF such as filgrastim and pegfilgrastim [17] are one of the most commonly reported medications that are associated with SS. Counterintuitively, some immunosuppressants like azathioprine [18], and immunomodulators have been reported to induce Sweet syndrome. For example, adalimumab was reported to induce SS in patients with hidradenitis suppurativa [19] and ulcerative colitis [20] but was successfully used in treating recalcitrant SS with a dramatic, robust clinical response [21]. The same disparity and paradoxes exist in reports about infliximab [18,22] and tocilizumab [23]. Recent research suggests that the neutrophilic dermatoses could be cutaneous expressions of autoinflammation through an aberrant hyperproduction of interleukin-1 [7], which was further supported by the sporadic reports on the efficacy of etanercept [24] and anakinra [25] in treating recalcitrant SS.

Sweet syndrome is considered a paraneoplastic disorder and approximately 21% of the patients have an associated malignancy. Sweet syndrome can precede, concur with, or follow the onset of malignancy, and may be the first sign of malignancy or its recurrence [26]. Fifty-three percent of uncategorized patients with Sweet syndrome were diagnosed with malignancy subsequently, up to 19 months later [27]. An intriguing topic is how to differentiate malignancy-associated versus non-malignancy-associated SS based on clinicopathological characteristics. A retrospective case-control study showed that the hemoglobin and platelet levels were significantly lower in malignancy-associated SS but no histopathological differences were observed [28]. Moreover, malignancy-associated SS was also characterized by the absence of arthralgia and histiocytoid histopathology, as it was reported that the finding of ulceration resembling pyoderma gangrenosum most commonly occurred in the setting of hematologic malignancy [26]. Moreover, the clinical presentation of SS can overlap with dermatological manifestations of hematological malignancy, such as leukemia cutis and mycosis fungoides, especially in the context of newly-emerging skin lesions with active hematological cancers [5]. This emphasizes the importance of further screening and continuous monitoring, which facilitates early detection of a potential underlying malignancy and assists in initiating adequate therapy. Other studies suggest that higher ESR elevation is associated with malignancy. However, considering the non-specific nature and expected ESR elevation in the context of acute inflammation, the association is questionable.

## Conclusions

Our case emphasizes the importance of keeping a high index of suspicion for SS when assessing the clinical constellations of fever, neutrophilia, and erythematous plaques suggesting atypical cellulitis. Due to the lack of a systematic approach to patients with SS, under-investigation and diagnostic delays are common. Even though hemoglobin and platelet levels were significantly lower in malignancy-associated SS, there is no specific marker that can definitely differentiate malignancy from non-malignancy-associated SS. Careful consideration of risk factors, red flag symptoms, and appropriate cancer screening should be employed.
